# Transesterification of Yellow Oleander seed oil, its utilization as biodiesel and performance evaluation

**DOI:** 10.1016/j.heliyon.2022.e09250

**Published:** 2022-04-08

**Authors:** Subhrajyoti Bhattacharyya

**Affiliations:** Deysarkar Centre of Excellence in Petroleum Engineering, IIT Kharagpur, West Bengal, India

**Keywords:** Biodiesel, Transesterification, Yellow Oleander seed oil, Soxhlet extraction method, GC-MS, FTIR

## Abstract

Traditional fossil fuels are our primary source of energy, but due to the rapidly increasing human population and their never-ending demands is diminishing the petroleum reserves quickly as they are in a limited stock inside the earth and the pollution caused by fossil fuels is a matter of great concern, so we need an alternative safe, clean & green source of fuel. Biodiesel is attracting everyone's eyes as an alternative and renewable energy. In this study, feedstock was prepared, and the oil was extracted from Thevetia peruviana seeds and transesterified. The transesterified biodiesel oil's physical and chemical properties were determined and compared with the universal standard values. The GC-MS and FTIR were used to determine fatty acids and esters present in the transesterified biodiesel oil. The novelty in this study is that the use of this novel method which produces an outstanding quality of Biodiesel oil, the methods employed in the analysis and determination of physicochemical properties and the chemical structure of the Thevetia Peruviana Biodiesel Oil and comparing these properties to check its usability as Biodiesel and the new type of non-edible oilseeds (Yellow Oleander) seeds used as a source of the biodiesel oil.

## Highlights

• Yellow Oleander seeds were collected, and their oil was extracted.
• The oil was transesterified, and its physicochemical properties were determined.
• The oil properties were compared with the properties of Biodiesel, Diesel.
• The oil properties were compared with the properties of B10, B20.

## Graphical abstract

**Figure:**
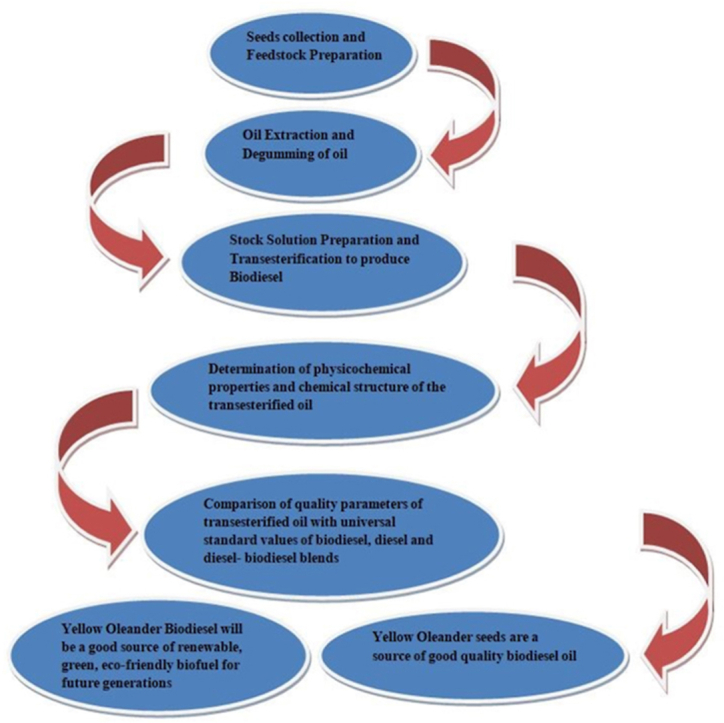

## 1 Introduction

Due to a significant increase in population and rapid economic development, the energy demand is increasing rapidly. The primary energy source in the world is conventional petroleum energy, coal, and natural gases. Fossil fuels contribute to a large extent to global warming, and these energy reserves are decreasing day by day, which will soon cause a shortage of power throughout the world. Fossil fuels cause a lot of pollution on burning and release many harmful gases on combustion; to prevent this, we need an alternative safe, clean & green source of fuel. Biodiesel is attracting everyone's eyes as an alternative and renewable energy for diesel engines. Biodiesel as an alternative and renewable fuel for diesel engines produces high cetane number, lower emission, possesses high flash point (FP), better lubrication, and excellent fuel properties. It does not increase the ${\text{CO}}_{2}$ level in the atmosphere, which in turn helps in the reduction of pollution. It is meant to be used in standard diesel engines. It can be used alone or blended with petrol diesel in any proportion. It can also be used as heating oil (Ana Godson and Udofia Bassey, 2015).

In this study, (Yellow Oleander) seed was selected because it is a new seed; it is not used in any work, so instead of throwing it away, it was decided to use it in work. It is non-edible, so there is no problem in using it. It has high oil content due to its non-involvement in any job. It is usually wasted. So it can be used to produce biodiesel as a clean and green energy provider as an alternative to traditional conventional sources of energy which are decreasing in stocks due to never-ending human demands.

## 2 Materials and equipment required

Thevetia Fruits, Knife, Hand-Powered Bench Grinder, Oven, Soxhlet Apparatus, n-Hexane, Mixture Of Toluene and Ethanol in the Ratio of 7:3 (L/L), Thimble, Round Bottom Flask, Distilled Water, Cotton, Water Bath, NaOH Pellet, Ethanol, Pyknometer, Rheometer, Bomb Calorimeter, Pensky Martens Closed Cup Tester, Rotary Evaporator, Ultrasonic Bath, Gas Chromatography-Mass Spectrometry (GC-MS) Apparatus, Fourier Transform Infrared Spectroscopy (FTIR) Apparatus.

## 3 Methodologies

### 3.1 Feedstock preparation

**Figure 1:**
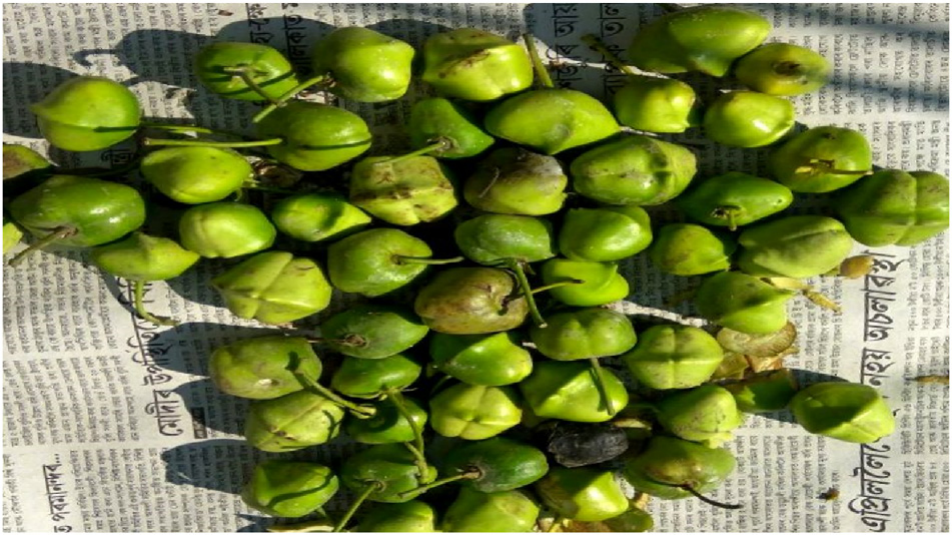
Thevetia Fruits.

The harvested Thevetia fruits were manually decorticated with a knife to reveal the kernels, as shown in Figure 1 through 2. Thevetia kernels were sundried for several hours continuously to ease the removal of the seeds from them. After the seeds were removed from the kernels, they were milled using a hand-powered bench grinder. The milled seeds were again sundried and subjected to many hours of the continuous oven-drying period until moisture contents were removed entirely, as shown in Figure 3. After these procedures, a constant weight was gained.

**Figure 2:**
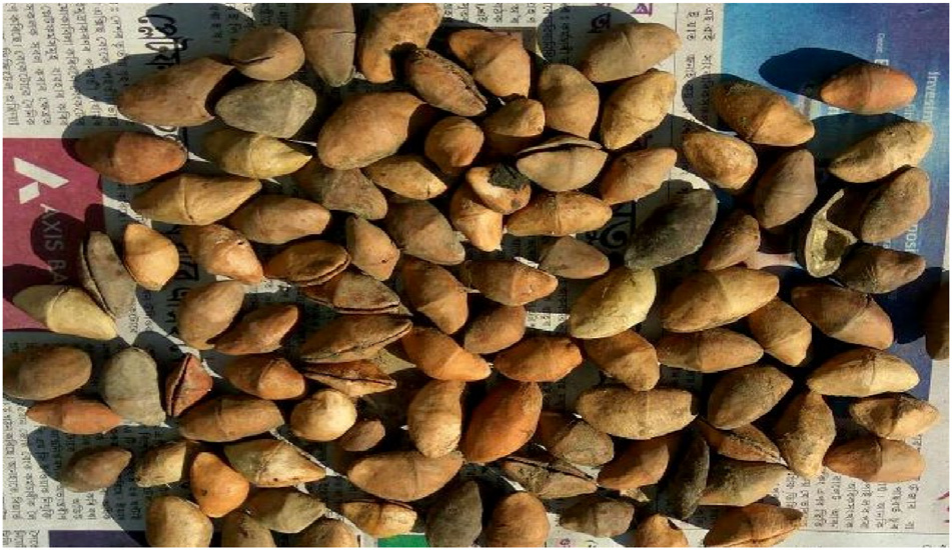
Thevetia Kernels.

**Figure 3:**
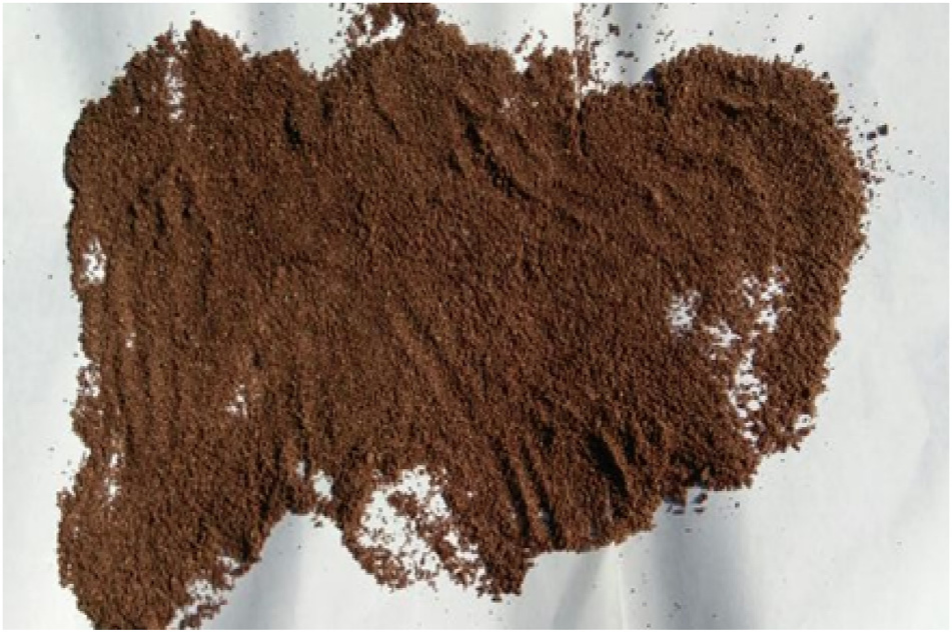
Sundried Milled Thevetia Seeds.

### 3.2 Oil extraction

The milled oven-dried samples were used for oil extraction. The method used for the extraction of oil was Soxhlet Extraction Method. Two different solvents were taken with two different samples to carry out the process. Solvents used were n-hexane and a mixture of toluene and ethanol in the ratio of **7:3 L/L**.

**Figure 4:**
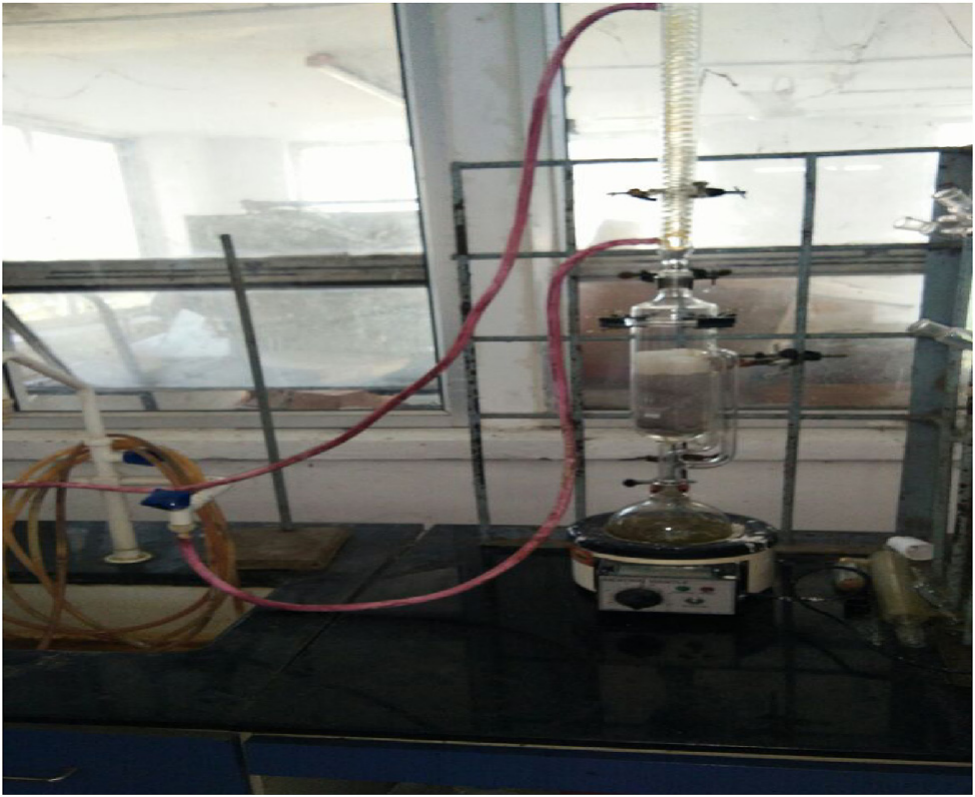
Soxhlet extraction process.

The weight of oven-dried milled seeds was measured in a macro meter and filled in a thimble, which comprises semi-permeable filter papers. The samples with thimble were placed in a Soxhlet. The solvent was taken in a round bottom flask, fitted in the bottom side of the Soxhlet. A Condenser was held at the upper side of the Soxhlet. The whole setup was placed in a Heating Mantle vertically, as shown in Figure 4. The round bottom flask was heated above the boiling point of the solvent used. The extraction processes were carried out for almost 12 h with respective solvents. The oil was extracted with the solvent in the round bottom flask.

The extracted oil with solvent in a round bottom flask was fitted in the rotary evaporator (Rotavapor) and was rotated with a uniform r.p.m. A vacuum was created inside the rotavapor, and after a certain period, the solvent was separated from the oil. The percentage of the oil content was determined by Eq. (1), as shown below.

(1) $$\text{Oil ​Content ​}\left(\text{\%}\right)=\frac{W_{o}}{W_{u}}\times 100\text{\%}$$

Where, **W**_**o**_ = weight of oil extracted (g)

**W**_**u**_ = weight of oven-dried milled seeds used for extraction process (g)

### 3.3 Degumming of the extracted oil

For degumming of the extracted oil, 90 ml of oil was mixed with 200 ml of distilled water in a separating funnel. The mixture was agitated slightly and allowed to settle for about 20 min. The precipitate was formed because of the mixing of water with gums.

**Figure 5:**
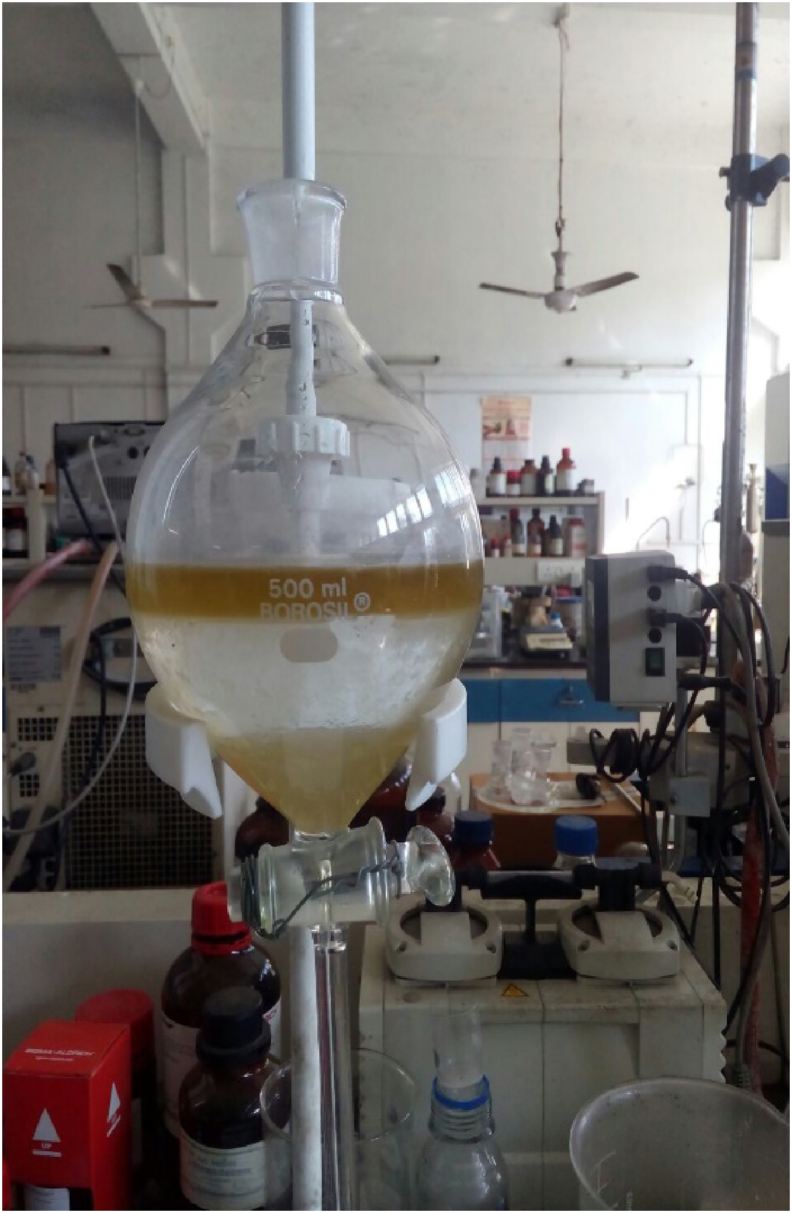
Oil-water mixture with gum.

**Figure 6:**
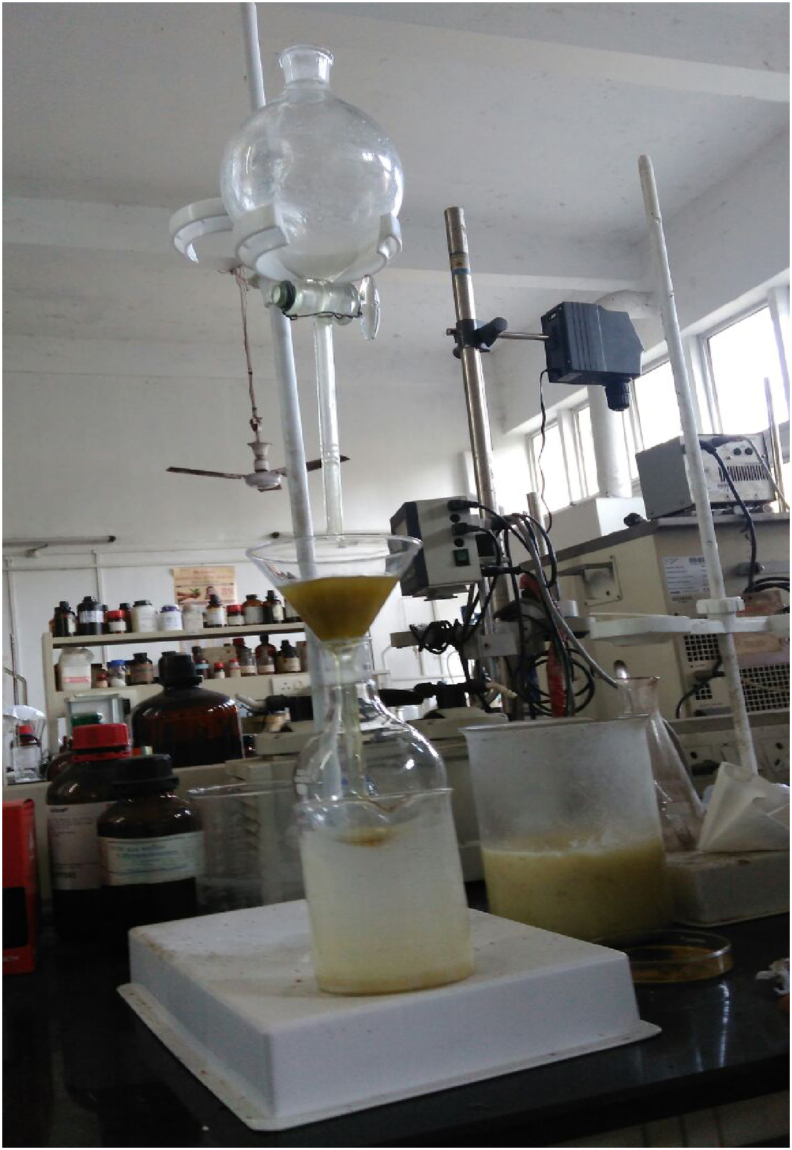
Degumming of oil.

Two separate layers were formed; the lower layer being the water-gum layer and the upper layer being the oil-gum layer, as shown in Figure 5. The water-gum mixture was drained off from the bottom of the separating funnel. To wash the oil gum layer, a funnel fitted with cotton was placed on the mouth of a round bottom flask. The remaining portion of the mixture was allowed to pass through the funnel equipped with cotton, as shown in Figure 6. Degummed oil with a small amount of water was extracted in the round bottom flask, which was again heated in a water bath over 100 °C to evaporate the water from the oil.

### 3.4 Transesterification of oil

**Figure 7:**
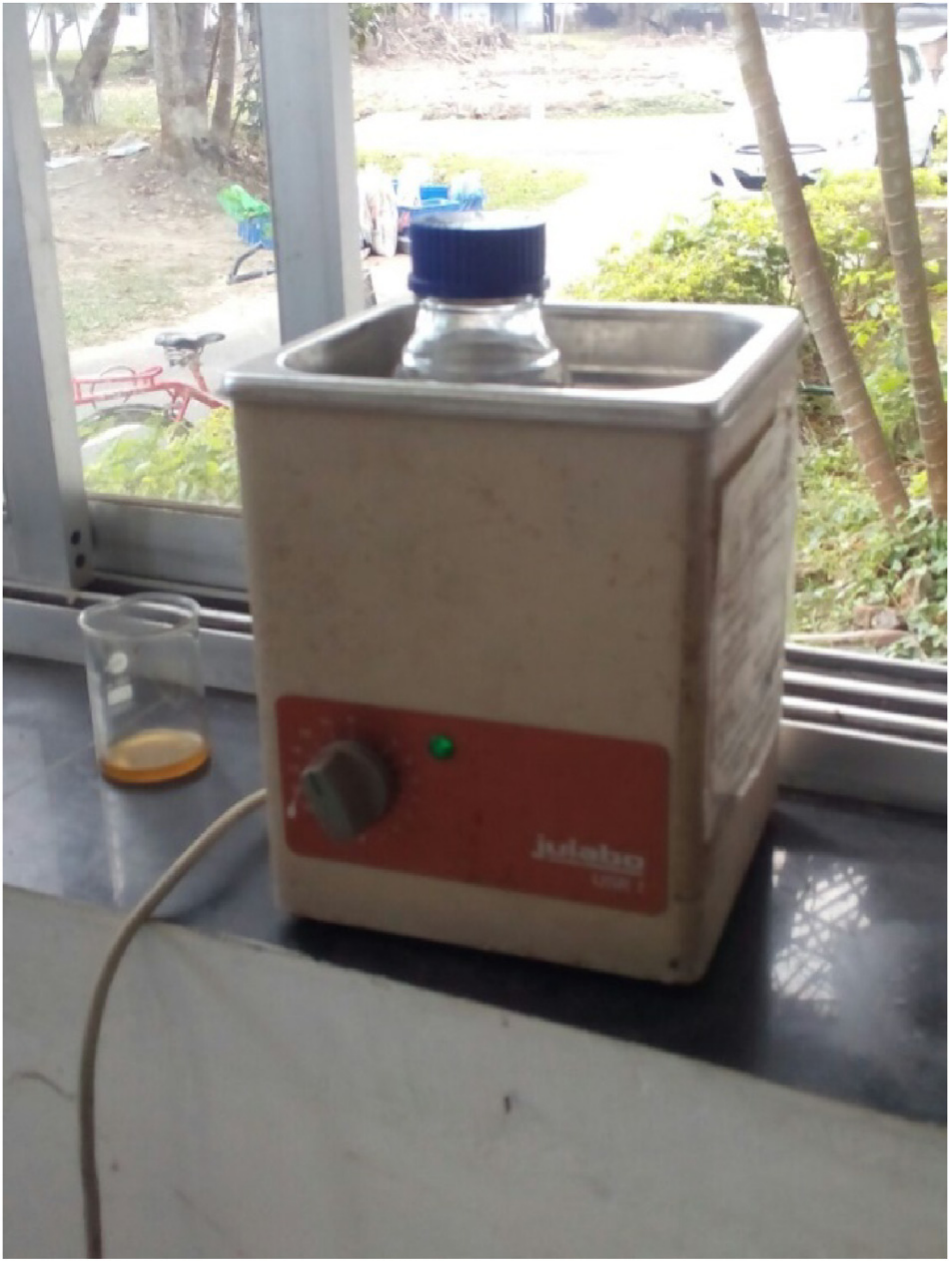
Preparation of stock solution in the ultrasonic bath.

100 g of methanol was mixed with 5 g of NaOH pellet to prepare a stock solution. The solution with apparatus was placed in an ultrasonic bath to dissolve the NaOH pellets in methanol, as shown in Figure 7. It was again placed in a refrigerator to dissolve completely.

**Figure 8:**
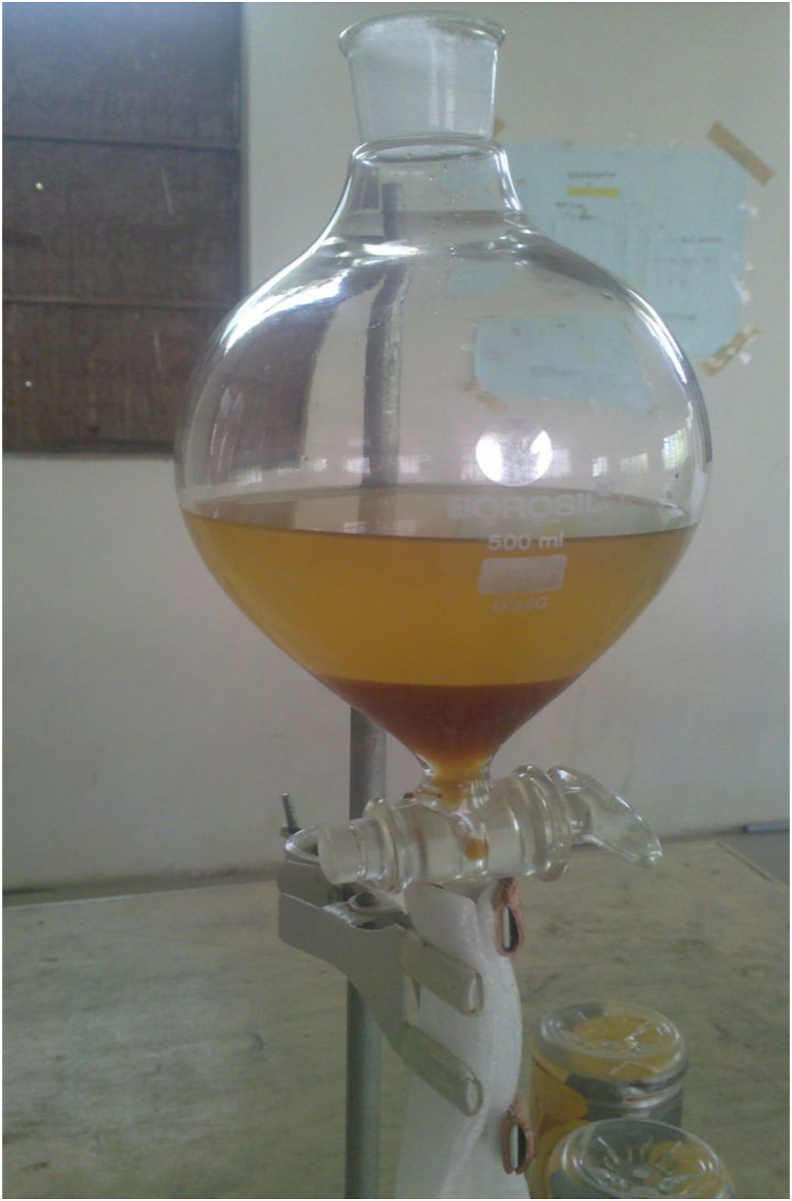
Transesterification process.

30 ml oil was taken for the transesterification process, as shown in Figure 8. The oil was mixed with 50 ml of stock solution in a round bottom flask and agitated for a couple of mins. NaO ${\text{CH}}_{3}$ present in the stock solution reacted with the oil to form glycerol, which settled in the bottom of the funnel. Glycerol was then separated to get the transesterified oil.

## 4 Results

### 4.1 Oil extraction

Table 1 shows the percentage of oil extracted from oven-dried milled yellow oleander seeds using n-hexane and a mixture of toluene and ethanol in the ratio of **7:3 (L/L)** as solvent.

**Table 1:** Calculation of oil extracted using different solvents.

Sample No.	Type of Solvent Used	Weight of Oven-Dried Milled Seeds (kg)	Weight of Oil Extracted (kg)	Oil Content (%)
1.	n-hexane	0.2587	0.14564	56.29
2.	n-hexane	0.2605	0.14372	55.17
3.	n-hexane	0.2563	0.1353	52.78
4.	n-hexane	0.2654	0.1404	52.90
5.	Mixture of toluene and ethanol	0.2889	0.1513	52.37

### 4.2 GC-MS spectrums

**Figure 9:**
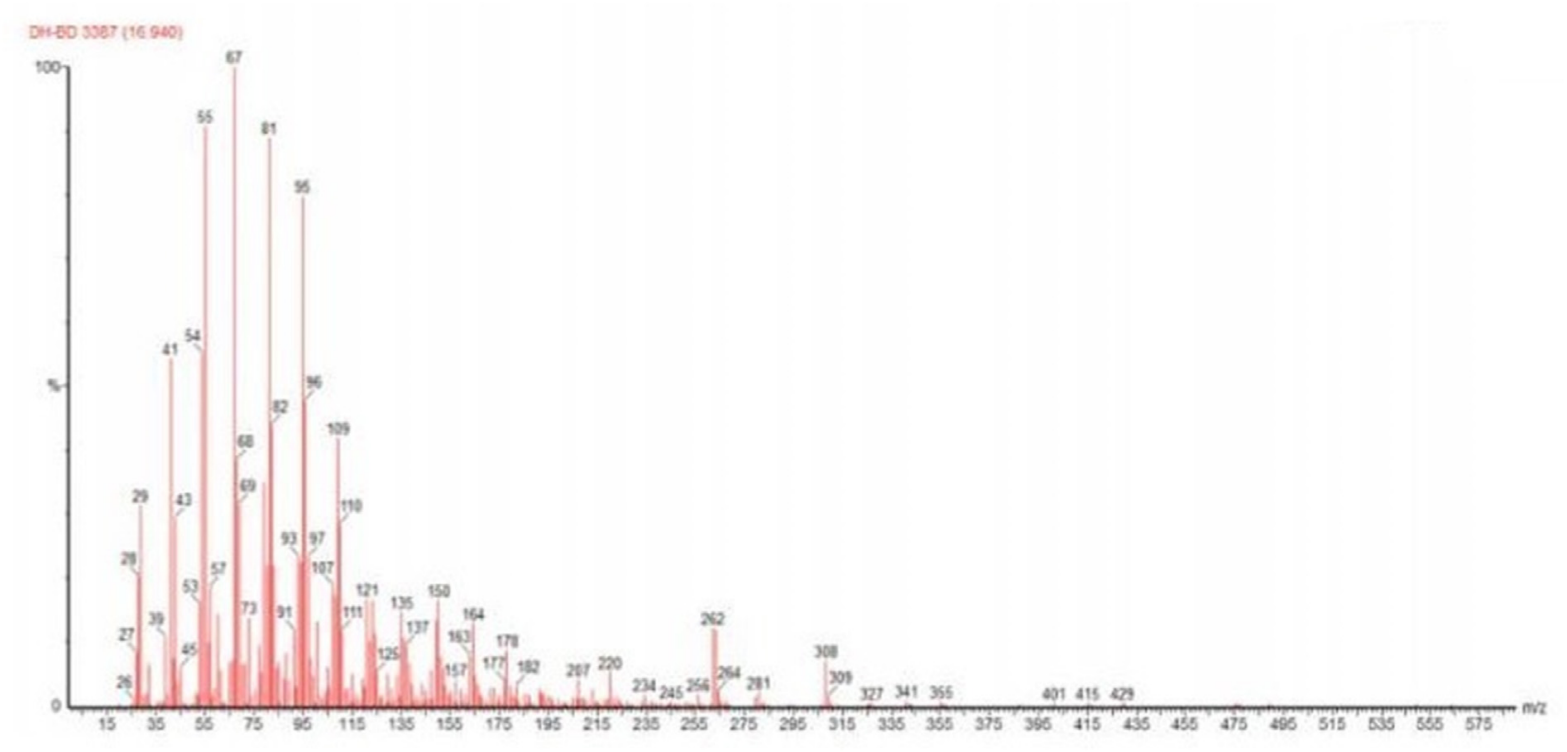
Spectrum of Methyl Linoleate present in Thevetia peruviana Biodiesel methyl ester.

Figure 9 through 10 show the GC-MS spectrum of Thevetia Peruviana Biodiesel methyl ester, showing the presence of Methyl Linoleate and Methyl Oleate.

**Figure 10:**
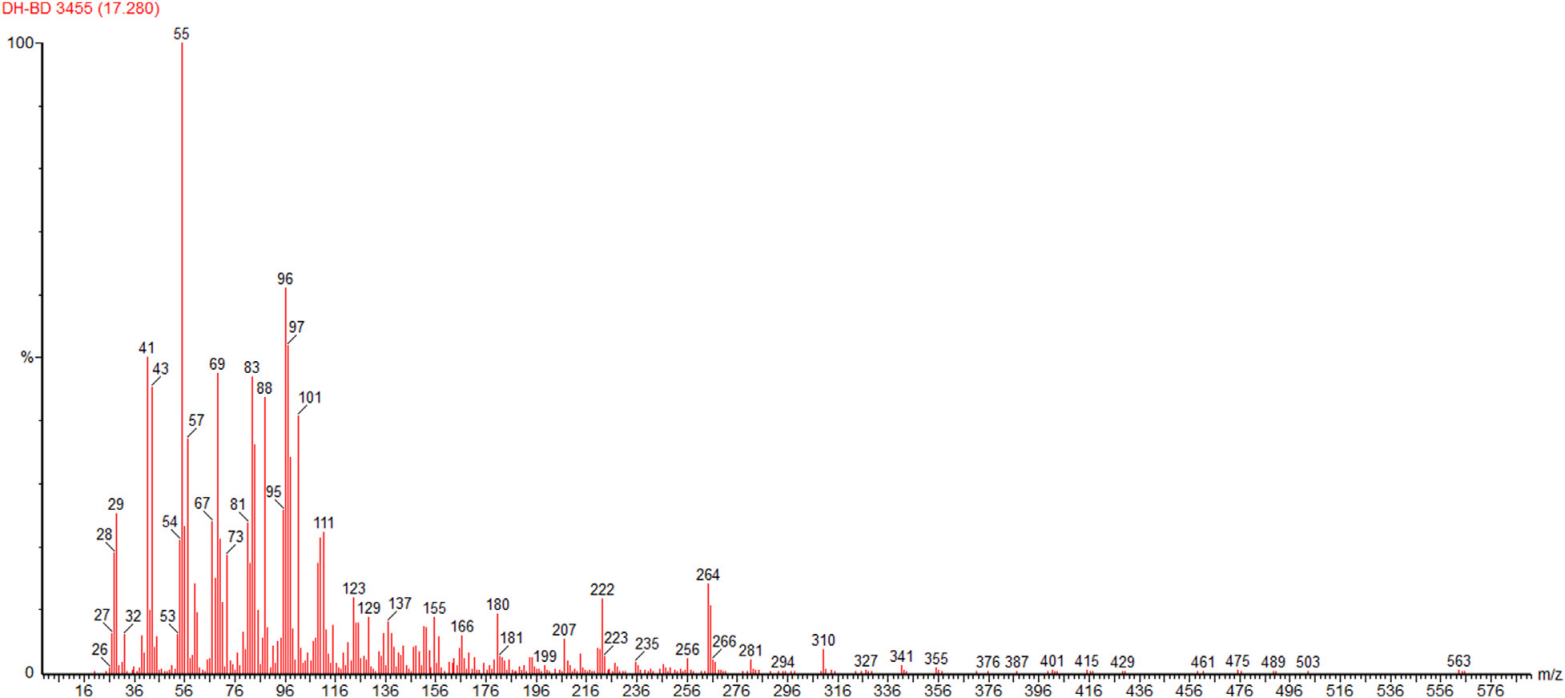
Spectrum of Methyl Oleate present in Thevetia peruviana Biodiesel methyl ester.

After studying the above two spectrums, the fatty acids present in Thevetia peruviana biodiesel methyl ester are mentioned in the Table 2 with IUPAC (International Union of Pure and Applied Chemistry) name along with the chemical formula.

**Table 2:** Fatty Acids present in Thevetia Peruviana Biodiesel.

Acids	IUPAC Name	Chemical Formula
Oleic Acid	Cis-9-Octadecanoic acid	C_18_H_34_O_2_
Palmitic Acid	Hexadecanoic acid	C_16_H_32_O_2_
Linoleic Acid	Cis-9,cis-12-Octadecanoic acid	C_18_H_32_O_2_
Stearic Acid	N-Octadecanoic acid	C_18_H_36_O_2_

### 4.3 FTIR spectrums

**Figure 11:**
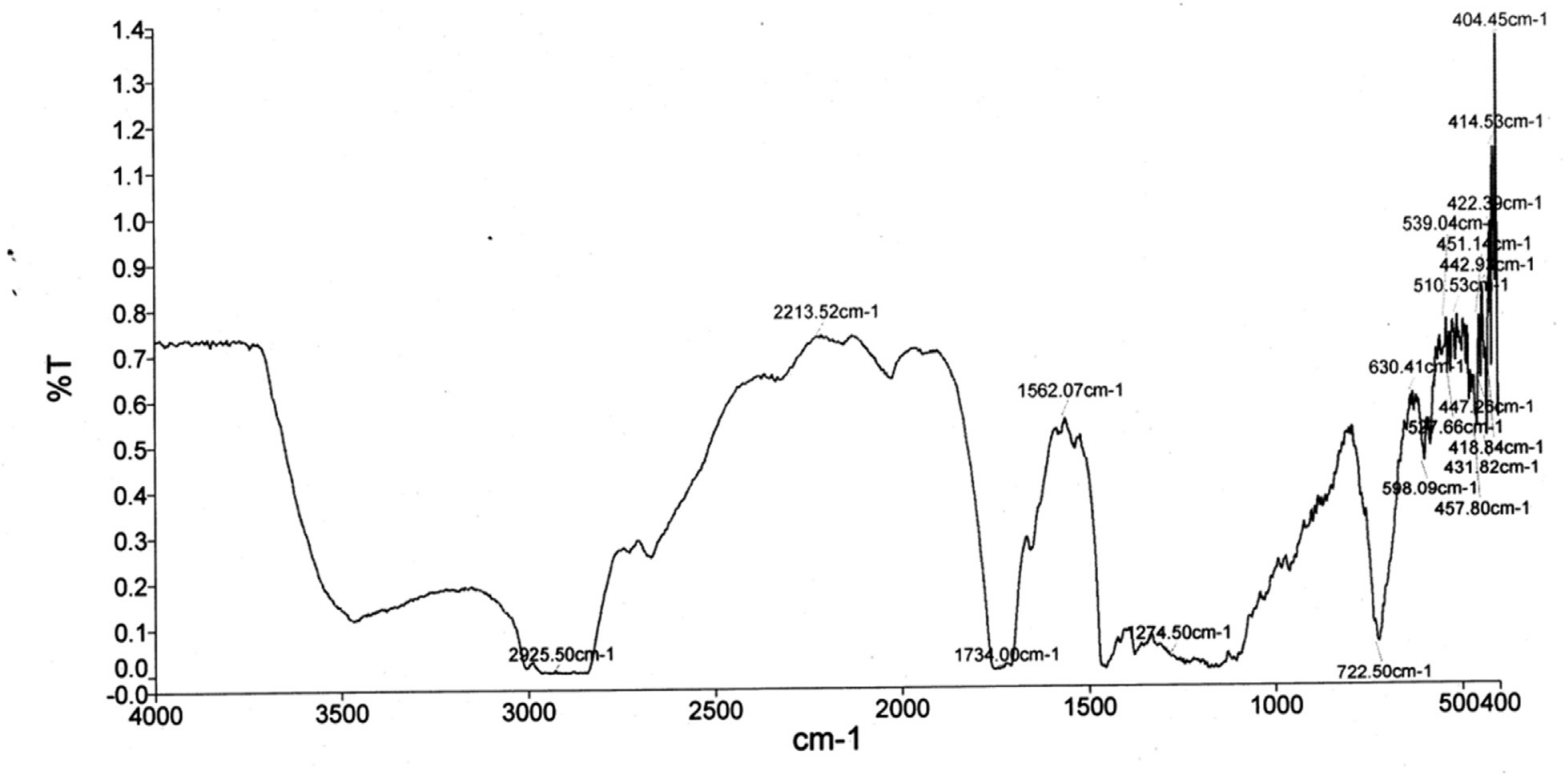
FTIR spectrum for ester in non transesterified oil.

Figure 11 through 12 show the FTIR spectrum of ester in non transesterified oil and biodiesel.

**Figure 12:**
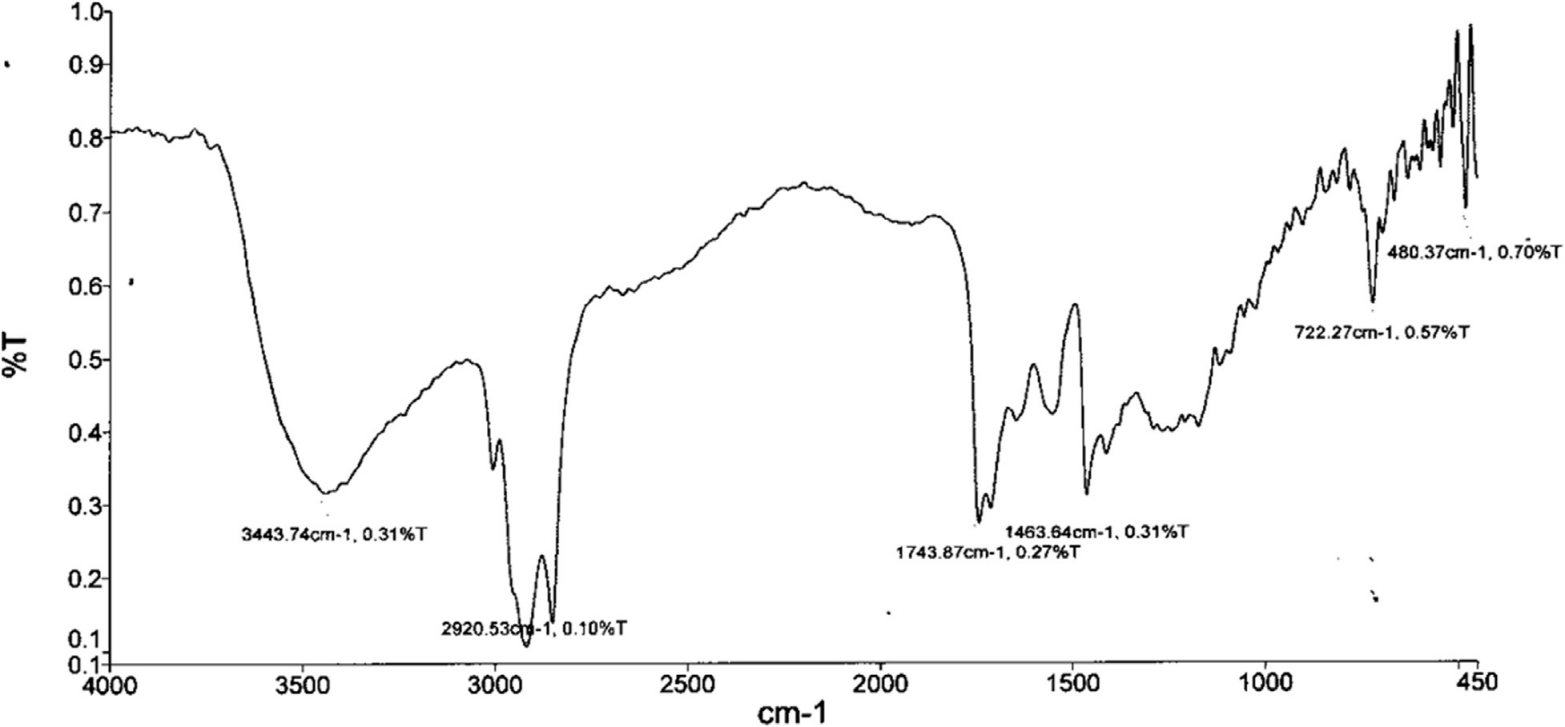
FTIR spectrum for ester in biodiesel.

### 4.4 Comparative analysis between diesel, *Thevetia Peruviana* biodiesel and its various blends

Table 3 through 4 show the comparison of quality parameters of Thevetia Peruviana Biodiesel with biodiesel standard and diesel and with diesel-biodiesel blend.

**Table 3:** Quality parameters of Thevetia peruviana biodiesel in comparison with bio-diesel standard and diesel.

Parameter	TP Biodiesel∗	Biodiesel Standard	Diesel
Density (kg/L) (at 40 °C)	0.853	0.860–0.890	0.820–.850
Viscosity (Pa.s) (at 40 °C)	5.6 × 10^−3^	(1.9–6.0) x10^−3^	(1.9–4.1) x10^−3^
Kinematic Viscosity (m^2^/s) (at 40 °C)	5.6	1.9–6.0	1.9–4.1
Flash Point (^o^C)	132	100–170	60–80
Pour Point (^o^C)	-3	-15 to 10	-35 to -15
Calorific Value (kJ/kg)	42636	37700	43400–44800

∗TP Biodiesel – Thevetia peruviana Biodiesel.

**Table 4:** Quality parameters of Thevetia peruviana biodiesel in comparison with diesel- biodiesel blend.

Parameter	TP Biodiesel∗	B20	B10
Density (kg/L) (at 40 °C)	0.853	0.848	0.847
Viscosity (Pa.S) (at 40 °C)	5.6 × 10^−3^	4.1 × 10^−3^	4.1 × 10^−3^
Kinematic Viscosity (m^2^/s) (at 40 °C)	5.6	4.1	3.9
Flash Point (^o^C)	132	84	79

∗TP Biodiesel – Thevetia peruviana Biodiesel.

## 5 Discussions

Shereena and Thangaraj (2009) found most of the existing literature is on edible oils. Very little literature represents non-edible oils. The mechanical characteristics of Biodiesel are equivalent to that of diesel, and it falls within the fuel standards and reduces engine emissions. Those obtained from non-edible oils are cheaper than that from edible oils. So, Biodiesel is a green, clean, renewable fuel for future generations.

For biodiesel production, Mohanty and Mishra (2013) utilized non-edible oils from Karanja, Jatropha, Sima-rouba, Mahua & Polanga, whose quality parameters values fall within the ASTM standards. We can conclude that non-edible oils are a good source of biodiesel production.

Kargbo (2015) studied biodiesel production from waste/used cooking oil by transesterification reaction in which methanol was used as a reactant, and KOH was used as a base catalyst. He found that all the quality parameters values fall within the ASTM standards and Exhaust particulate matter (PM) emissions of Biodiesel are much lower than fossil fuels.

Chhetri et al. (2008) used oil-bearing plants like soapnut (Sapindus mukorossi) and jatropha (Jatropha curcas, L.) as the feedstock for biodiesel production. He found that the oil from these plants has a great potential to be used as a source for biodiesel production. The biodiesel produced from these plants is environment-friendly, and their quality parameters fall within the ASTM standards. It can be a good source of renewable, cleaner, safer, eco-friendly biofuel for future generations.

Munir et al. (2021) utilized Raphnus raphanistrum L. seed oil to produce biodiesel as an alternative to traditional edible seed oils, which is not, encouraged due to the ongoing food shortage and consequent shortage price hikes. It was observed that Raphnus raphanistrum L. seed is not only a promising feedstock for producing biodiesel, but it produces a good yield and quality of biodiesel whose quality parameter values lie within the ASTM standards.

Vijayaram (2021) prepared biodiesel from edible waste oil like rice bran and sunflower oil by transesterification. He found that the quality parameters of Biodiesel were very excellent and within the ASTM standard. It can be directly employed in modern diesel engines without appreciable loss in engine performance.

Demisu (2021) found that Biodiesel can be produced from edible oils by various established chemical processes. The production from these processes requires good technical knowledge and skills of the different operational variables to have a good yield and superior quality of biodiesel oil.

Thilakarathne et al., (2021) found that waste cooking oil from local restaurants is a cost-effective source of biodiesel production, and it helps prevent environmental pollution. It will also prevent the reuse of waste cooking oil by street sellers as the reuse of waste cooking oil acts as a carcinogenic or cancer-causing enzyme. So, the reuse of waste cooking oil to produce biodiesel will also protect human health.

Shaah et al. (2021) found that non-edible oils are a good source of quality biodiesel. Its use will reduce the cost of biodiesel production as humans do not consume non-edible oils due to the presence of toxic components. It will also provide an alternative to edible oils for the benefit of biodiesel production, which is costly as humans consume them. We can grow non-edible oil plants in harsh and non-fertile lands, *i.e.,* it doesn't require any agricultural land for growth, so it will further save the cost incurred on making land fertile and suitable for agriculture.

El-gharbawy et al. (2021) found that biodiesel production from non-edible oils is cost-effective and efficient. The alkaline catalyzed transesterification method is the most common process for biodiesel production. The utilization of biodiesel will significantly affect the environment, vehicle engines, independence on crude oil, investment, and the economy as it will create jobs.

It was observed that non-edible oils like Yellow Oleander (Thevetia Peruviana) seed oil are a good source of cheaper and quality biodiesel oil than edible oils. The novelty in this study is that the use of this novel method which produces an outstanding quality of Biodiesel oil, the methods and techniques employed in the analysis and determination of physicochemical properties and the chemical structure of the Thevetia Peruviana Biodiesel Oil and comparing these properties with the Biodiesel Standard, diesel, and diesel-biodiesel blends to check its usability as Biodiesel and the new type of non-edible oilseeds (Yellow Oleander) seeds used as a source of the biodiesel oil.

The use of Yellow Oleander seeds for biodiesel production is economically viable because the operating costs of all the equipment are significantly less, the types of equipment and solvents used here are readily available in a chemical laboratory and are cheap, yellow oleander seeds are readily available in abundant quantities and all the methods explained here from extraction of oil to comparison of properties are very simple and can be done by simple equipment and solvents which are generally available in the chemical laboratory. The biodiesel produced by this method is very cheap economically viable, and its excellent quality parameters boost its economic viability.

In the future, we can undergo further research to employ innovative solvent compositions and methods to extract more % of oil from the feedstocks and produce the biodiesel at a mass scale that can benefit a considerable population and the biodiesel production process becomes cheap. To search more other non-edible seeds in more abundant quantities like the one used here so that the biodiesel production process doesn't interfere with the eating source of people and at the same time we can save the fossil fuels reserves which are rapidly depleting and are in a limited stock inside the earth and protect the environment from the damaging effects of fossil fuel usage to a large extent as possible.

## 6 Conclusions

In the Soxhlet Extraction Method, when n-hexane was used as a solvent, it extracted more oil from milled oven-dried seeds than the mixture of toluene and ethanol as solvent and provided a better yield. The oil obtained from Thevetia peruviana seeds was considerably high concerning the feedstock quantity (average of 52 %), and the transesterification process proved to be an effective refining method. From the GC-MS Spectrums, it was observed that Oleic Acid, Palmitic Acid, Linoleic Acid, and Stearic Acid were present in Thevetia peruviana Biodiesel. At 40 °C, The Thevetia peruviana Biodiesel has a density of 0.853 kg/L, a viscosity of 5.6 × 10-3 Pa.s, kinematic viscosity of 5.6/s, a flashpoint of 132 °C, a pour point of -3 °C, and a calorific value of 42636 kJ/kg. These parameter values lie in the universal standard values of biodiesel and diesel. It has a calorific value more than biodiesel and diesel and was better than two diesel-biodiesel blends (B10 and B20). It can be concluded that Thevetia peruviana Biodiesel will be a good source of renewable, cleaner, safer, eco-friendly biofuel for future generations compiled with many benefits and can be an excellent alternative to traditional fossil fuels. This will save our environment from the damaging effects of fossil fuel usage and boost other sectors of our economy. It can also be concluded that Yellow Oleander (Thevetia Peruviana) seeds are a good source of quality biodiesel oil. This conforms to global expectations and will add to biodiesel resource infrastructure in the world's tropical region where Thevetia peruviana plants are found.

## Declarations

### Author contribution statement

Subhrajyoti Bhattacharyya: Conceived and designed the experiments; Performed the experiments; Analyzed and interpreted the data; Contributed reagents, materials, analysis tools or data; Wrote the paper.

### Funding statement

This research did not receive any specific grant from funding agencies in the public, commercial, or not-for-profit sectors.

### Data availability statement

Data included in article/supplementary material/referenced in article.

### Declaration of interests statement

The authors declare no conflict of interest.

### Additional information

No additional information is available for this paper.
